# Fine-grained classification based on multi-scale pyramid convolution networks

**DOI:** 10.1371/journal.pone.0254054

**Published:** 2021-07-09

**Authors:** Gaihua Wang, Lei Cheng, Jinheng Lin, Yingying Dai, Tianlun Zhang

**Affiliations:** 1 School of Electrical and Electronic Engineering, Hubei University of Technology, Wuhan, China; 2 Hubei University of Technology Cooperative Innovation Center of Hubei Province for Efficient Use of Solar Energy, Wuhan, China; Vellore Institute of Technology: VIT University, INDIA

## Abstract

The large intra-class variance and small inter-class variance are the key factor affecting fine-grained image classification. Recently, some algorithms have been more accurate and efficient. However, these methods ignore the multi-scale information of the network, resulting in insufficient ability to capture subtle changes. To solve this problem, a weakly supervised fine-grained classification network based on multi-scale pyramid is proposed in this paper. It uses pyramid convolution kernel to replace ordinary convolution kernel in residual network, which can expand the receptive field of the convolution kernel and use complementary information of different scales. Meanwhile, the weakly supervised data augmentation network (WS-DAN) is used to prevent over fitting and improve the performance of the model. In addition, a new attention module, which includes spatial attention and channel attention, is introduced to pay more attention to the object part in the image. The comprehensive experiments are carried out on three public benchmarks. It shows that the proposed method can extract subtle feature and achieve classification effectively.

## 1 Introduction

Fine grained image classification is a more and more concerned subject in the field of computer vision. It has been widely studied in new retail, automatic driving, ecological protection. Different from traditional image classification, fine-grained image classification aims to divide the same species into different subclasses, such as Shiba Inu and Akita Inu. Because most of the subtle differences between classes can only be distinguished effectively by region location and discriminative feature learning, fine-grained image recognition is regarded as a more challenging task.

Early works on localization-based methods usually use strong supervision to annotate the part information of the image, and then extract the features of the parts for fine-grained classification [1–3]. However, the methods of strong supervision rely heavily on manual object annotation, which is too expensive to be widely used in practice. Weak supervision using attentional mechanisms [4–8] have been more popular in subsequent researches. In [5], a recurrent attention convolutional neural network that locates the region of interest is proposed. By using the mutual promotion and enhancement of region detection and feature extraction, it gradually locates and identifies the region to complete the feature extraction from coarse-grained to fine-grained. In [6], it proposes to use pairwise interaction to distinguish differences and find the key areas of each image by comparing a pair of fine-grained images.

These methods all adopt the idea of location recognition, and strengthen the attention and discrimination to the fine features through the weak supervision method. However, they ignore the multi-scale features of the network. For fine-grained classification tasks, multi-scale features are crucial, because target parts have multifarious sizes and shapes in images [9–11], and the extracted feature of single convolution is insufficient.

This paper proposes a fine-grained classification network based on multi-scale pyramid convolution kernel, which uses pyramid convolution kernel with multiple sizes to extract multi-scale features. And weakly supervised data augmentation network (WS-DAN) is used to enhance the network data. To reduce the interference of image background, a new attention mechanism is also added to extract subtle features. The main contributions of this paper are as follows: (1) The pyramid convolution kernel is introduced to extract multi-scale features without increasing the computational cost, and improve the ability to capture fine features by WS-DAN. (2) A new lightweight multi-attention module, including spatial attention and channel attention, is designed to retain important information and suppress the interference caused by background noise. The attention module can be seamlessly integrated into any convolutional neural networks (CNN) architecture effectively (3) the proposed method achieves state-of-the-art performance on three datasets, including the CUB-200-2011 [12] dataset, Stanford Dogs [13] dataset, and Stanford Cars [14] dataset.

The rest of this paper is organized as follows: in Section 2, we introduce the Weakly Supervised Data Augmentation Network. Section 3 introduces the multi-scale pyramid convolution kernel and multi-attention module. Section 4 gives our experiment result, including the introduction about CUB-200-2011, Stanford Dogs, and Stanford Cars dataset, and the experiment settings. Finally, we conclude in Section 5.

## 2 Related work

### 2.1 Weakly supervised data augmentation network

Deep learning is an emerging technology in the field of machine learning, which has attracted the attention of many researchers [15–17]. Data enhancement is a common strategy in deep learning task. It can increase the number of training data by introducing more data variance in the way of random cropping. However, due to random sampling of clipping area, a large part of the area contains a lot of background noise, which may affect the quality of feature extraction and offset its advantages. Therefore, we use WS-DAN instead of traditional data enhancement.

WS-DAN uses attention learning to generate attention maps to represent the spatial distribution of the discriminating object parts, and then enhance the data of object. After generating the attention graph, the design is divided into two parts: the first part includes Attention cropping and Attention dropping. Attention cropping is the acquisition of subtle feature to enhance the representation of local features. Attention dropping removes subtle feature from the image randomly to learn new detail features. In the second part, the attention map is used to locate the whole target accurately, which enlarges the target to get more attention, and suppresses the interference of irrelevant noise.

### 2.2 Visual attention

Visual attention is widely used in various deep learning tasks [18–20]. It can be employed to discover the subtle inter-class differences in fine-grained image categorization. For instance, in [21], it proposes a cross layer non local module based on visual attention. By establishing a query layer and a response layer, the deep and shallow characteristics of the network are correlated to improve the representation ability of the network. The paper [8] uses the method of soft attention, which imposes a soft mask on the feature maps to generate the attention maps and guide the enhanced area of the image. The paper [22] uses a self-attention method to discover the complementary information related to channels through the interaction between channels.

## 3 The proposed method

**Fig 1:**
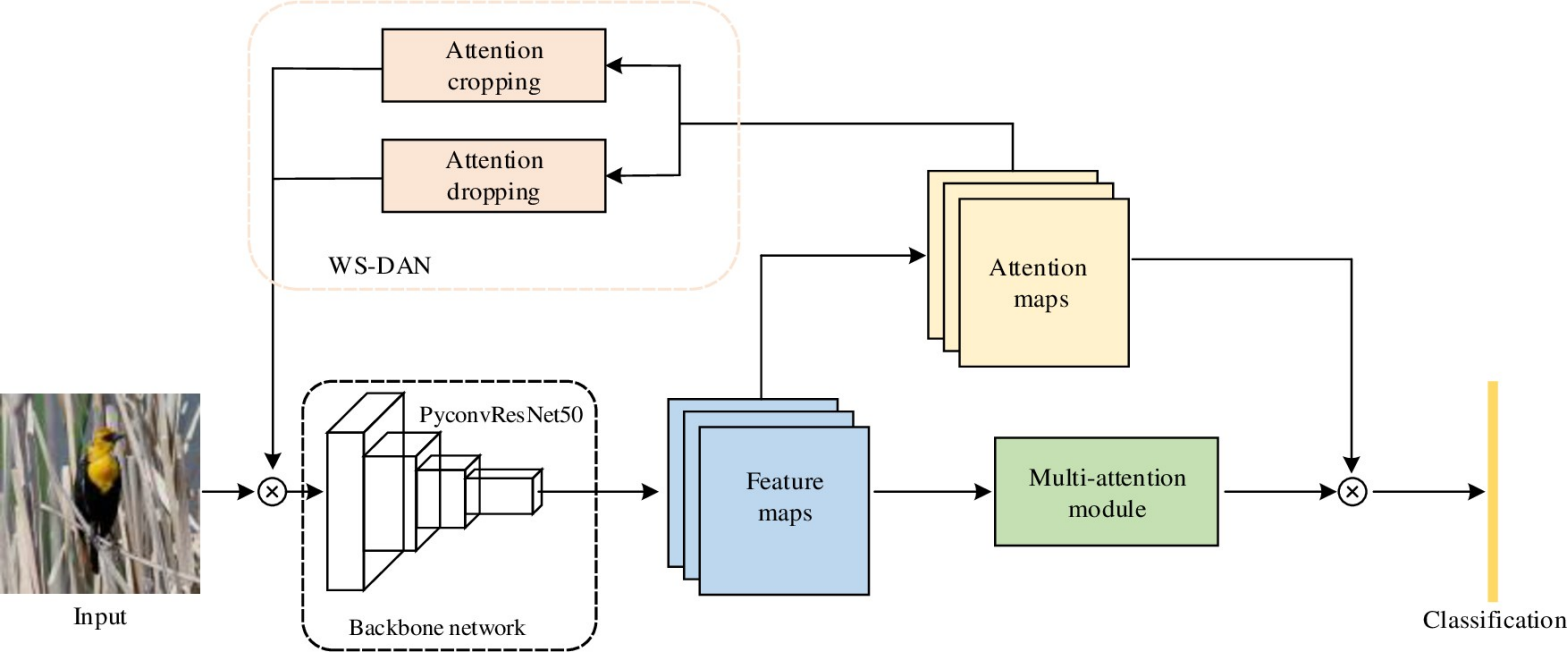
The overall architecture of the network.

The overall architecture of the network is shown in Fig 1. The backbone network is used to extract feature information, the multi-attention module is used to integrate spatial and channel information, and the weakly supervised data augmentation network (WS-DAN) is used to enhance image data and improve model performance.

The process of data passing through the network is as follows: first, the input image is sent into the backbone network to obtain feature maps. Then, attention maps are obtained through the convolution operation by feature maps, at the same time, feature maps are input into the multi-attention module. Output of attention maps are divided into two branches. One branch goes through the weakly supervised data augmentation network (WS-DAN) and feeds the results back to the input data; the other branch is fused with the output of the multi-attention module. Finally, the fused results are classified.

### 3.1 Multi-scale pyramid convolution

**Fig 2:**
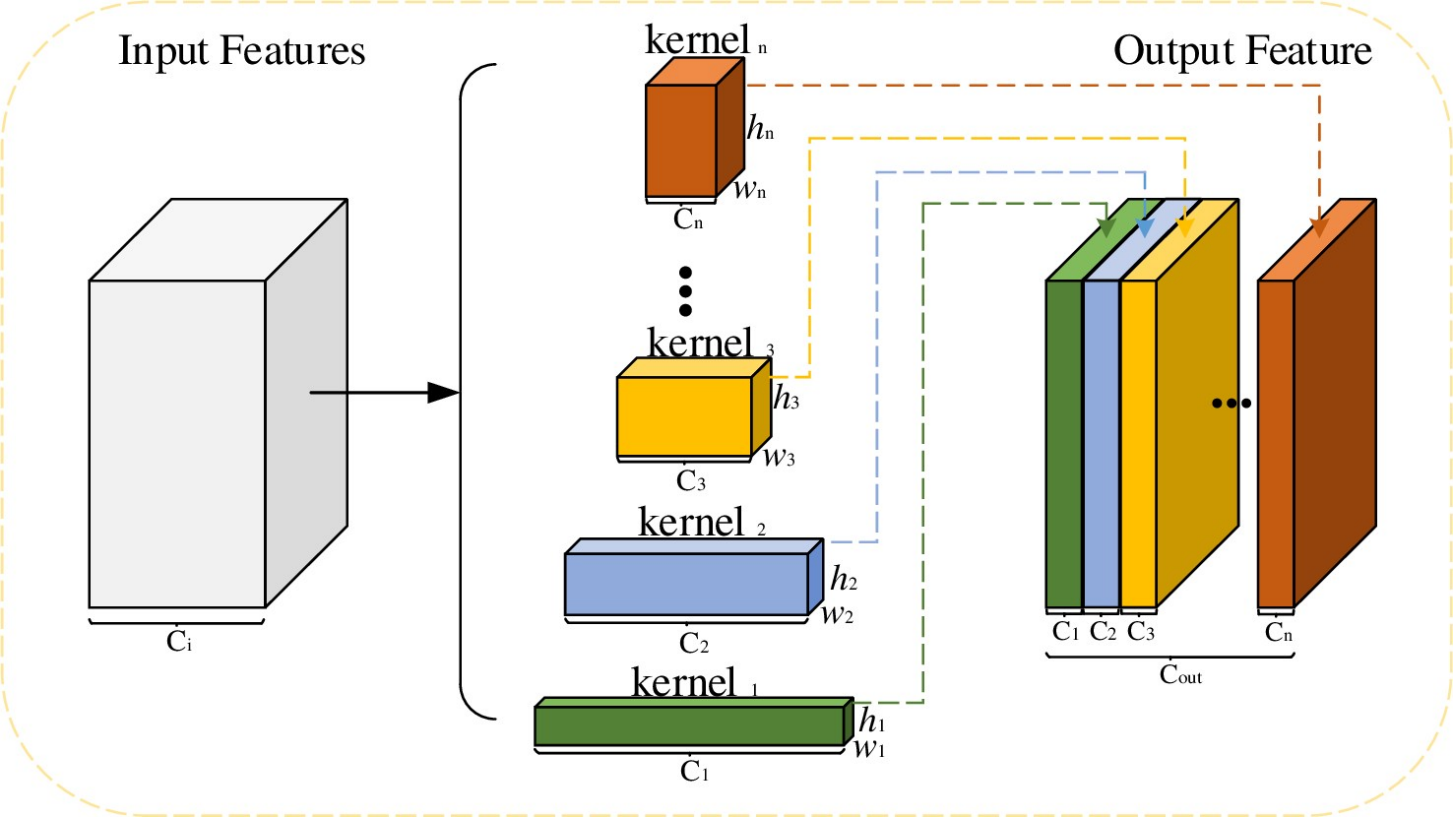
Multi-scale pyramid convolution kernel.

According to Convolutional Neural Networks (CNN) theory, the convolution operator can be transformed as follows: *T*:*X*→*Y*,*X*∈*R*^*h*×*w*×*C*^,*Y*∈*R*^*h*′×*w*′×*C*^, where *h*×*w* represents the spatial dimension and *C* represents the number of channels. Compared with ordinary convolution kernel, pyramid convolution kernel contains multiple scale convolution kernel. It can extract multi-scale features by using the convolution kernel of different sizes. Fig 2 is the structure of multi-scale Pyramid convolution (Pyconv).

In Fig 2, it contains several convolution kernels of different sizes (kernel_1_, kernel_2_,…, kernel_n_). The number of their output channels is *C*_1_,*C*_1_,⋯,*C*_*n*_, and the number of final channel is *C*_*out*_:

$$C_{out}=C_{1}+C_{2}+\cdots +C_{n}$$ (1)

Pyconv combines these features in channel dimensions to complete feature fusion, which helps the network obtain richer semantic information and locate the key areas of the image accurately.

**Fig 3:**
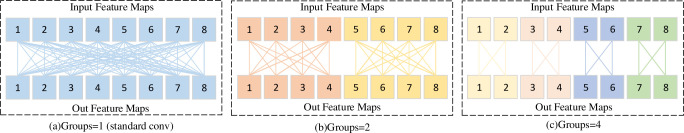
Grouped convolution.

In order to use different depth kernel in each level of Pyconv, the input feature maps are divided into different groups. As shown in Fig 3, they are three different groups: the numbers of groups are one group, two groups, and four groups. And the kernel is applied independently for each group, which is called block convolution. When the number of groups increases, the number of parameters and the calculation cost of convolution decrease.

The block convolution can reduce the parameters of the network. The total number of parameters of a convolutional layer is:

T(conv)=C×H×W×K (2)

Where *C* represents the number of channels, *H*×*W* represents the size of convolution kernel, and *K* represents the number of cores. In Table 1, the number of parameters in the first stage can be obtained through the following formula:

T(basic)=(64×3×3×64)×256×3=36864×256×3 (3)

T(PyConv4)=((64÷16×9×9×16)+(64÷8×7×7×16)+(64÷4×5×5×16)+(64÷1×3×3×16))×256×3=27072×256×3 (4)

**Table 1:** PyconvResNet50 convolution kernel size information.

Stage	Output	ResNet50(basic)	PyconvResNet50
	112×112	$\begin{array}{l} 7\times 7,t=64,s=2\\ 3\times 3,\max pool,s=2 \end{array}$	7×7, *t* = 64,*s* = 2
1	56×56	$\left[\begin{array}{c} 1\times 1,t=64\\ 3\times 3,t=64\\ 1\times 1,t=256 \end{array}\right]\times 3$	$[\begin{array}{c} 1\times 1,t=64\\ \underline{Pyvonv4,t=64}\\ \left[\begin{array}{ccc} 9\times 9, & t=16, & G=16\\ 7\times 7, & t=16, & G=8\\ 5\times 5, & t=16, & G=4\\ 3\times 3, & t=16, & G=1 \end{array}\right]\\ 1\times 1,t=256 \end{array}]\times 3$
2	28×28	$\left[\begin{array}{c} 1\times 1,t=128\\ 3\times 3,t=128\\ 1\times 1,t=512 \end{array}\right]\times 4$	$[\begin{array}{c} 1\times 1,t=128\\ \underline{Pyconv3,t=128}\\ \left[\begin{array}{ccc} 7\times 7, & t=64, & G=8\\ 5\times 5, & t=32, & G=4\\ 3\times 3, & t=32, & G=1 \end{array}\right]\\ 1\times 1,t=512 \end{array}]\times 4$
3	14×14	$\left[\begin{array}{c} 1\times 1,t=256\\ 3\times 3,t=256\\ 1\times 1,t=1024 \end{array}\right]\times 6$	$[\begin{array}{c} 1\times 1,t=256\\ \underline{Pyconv2,t=256}\\ \left[\begin{array}{ccc} 5\times 5, & t=128, & G=4\\ 3\times 3, & t=128, & G=1 \end{array}\right]\\ 1\times 1,t=1024 \end{array}]\times 6$
4	7×7	$\left[\begin{array}{c} 1\times 1,t=512\\ 3\times 3,t=512\\ 1\times 1,t=2048 \end{array}\right]\times 3$	$[\begin{array}{c} 1\times 1,t=512\\ \underline{Pyconv1,t=512}\\ \left[\begin{array}{ccc} 3\times 3, & t=512, & G=1 \end{array}\right]\\ 1\times 1,t=2048 \end{array}]\times 3$
	1×1	global avgpool fc	global avgpool fc
Params		25.56×10^6^	24.85×10^6^
FLOPs		4.14×10^9^	3.88×10^9^

Compared with ordinary convolution kernel, Pyconv kernel can reduce the number of parameters. we can get PyConvresnet50 by replacing the ordinary convolution kernel in the ResNet50 with multi-scale Pyconv kernel. The information of the convolution kernel of pyconvresnet50 is shown in Table 1, *s* represents the step size, *t* represents the number of channels, and *G* represents the channel group. The steps of PyConvresnet50 are as follows:

Step 1: The ordinary convolution is replaced by Pyconv4, which contains 9×9,7×7,5×5 and 3×3 kernels. The input channel of each group is *G* = 16, *G* = 8, *G* = 4 and *G* = 1, and the output channel of all kernels is 16.
Step 2: The ordinary convolution is replaced by Pyconv3, which contains 7×7,5×5 and 3×3 kernels. The input channel of each group is *G* = 8, *G* = 4 and *G* = 1, and the output channel is 64, 64 and 32;
Step 3: The ordinary convolution is replaced by Pyconv2, which contains 5×5 and 3×3 kernels, The input channel of each group is *G* = 4 and *G* = 1, and the output channel of all kernels is 128;
Step 4: Replace the original ordinary convolution kernel with Pyconv1 that only contains a convolution kernel 3×3. The number of output channels is 512, and the input channel is G = 1.

### 3.2 Multi-attention module

**Fig 4:**
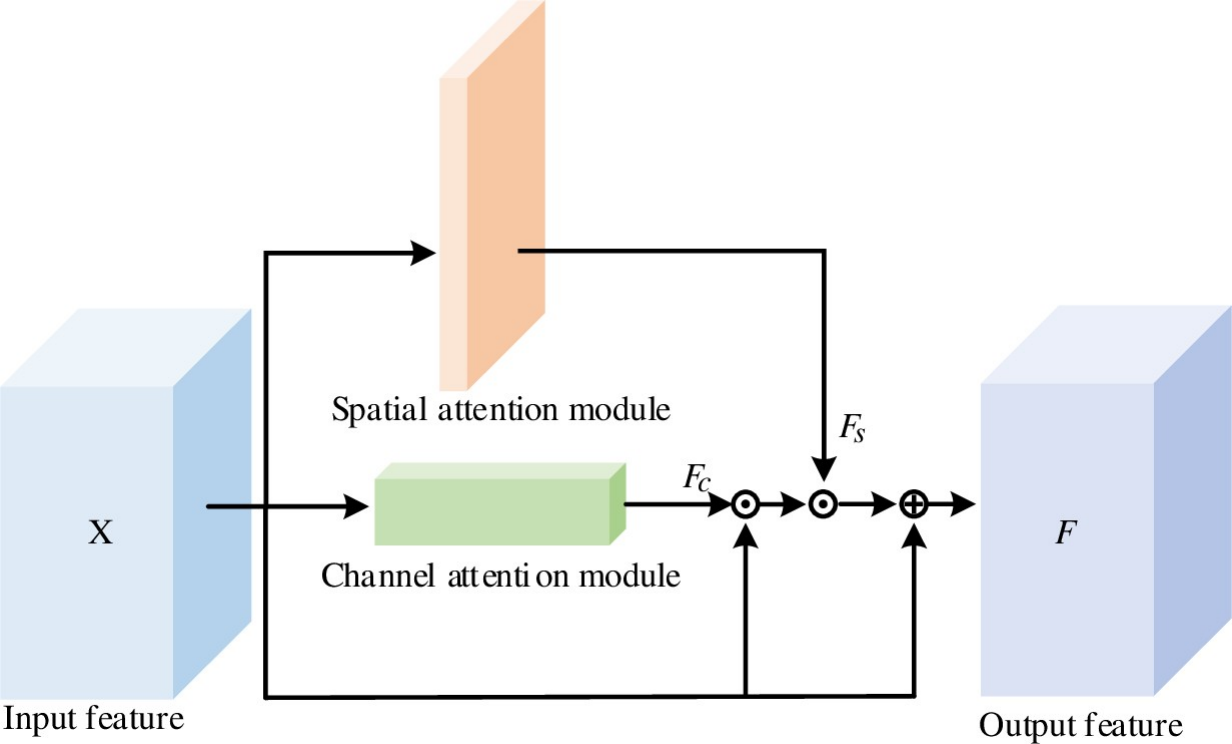
Multi-attention module.

To better extract the subtle features between different categories, a multi-attention mechanism, which includes channel attention and spatial attention, is designed. The structure of multi-attention module is shown in Fig 4.

The input X is paralleled through the channel and spatial attention to obtain the channel and spatial weight respectively. The network can learn the location information of the key area by multiplying with the channel and spatial weight to remove the interference of irrelevant background. Then the attention result is combined with the input feature X. It can be described as follows:

$$F=F_{s}*(F_{c}*X)+X$$ (5)

where *X* is input feature, *F* is output, *F*_*c*_ and *F*_*s*_ are output of channel attention and spatial attention respectively.

#### Channel attention module

**Fig 5:**
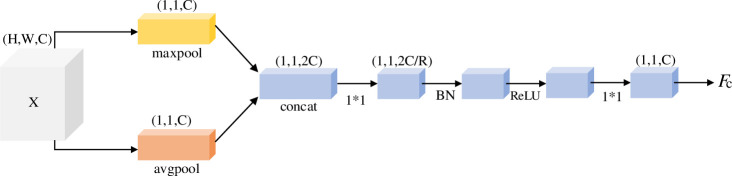
Channel attention module.

Channel attention can effectively capture the context information between channel information. Fig 5 shows the specific method of the channel attention module. Firstly, global maximum pooling and global average pooling are used to map input features from space (*H*,*W*,*C*) to (1,1,*C*). Then, the results of the two pooling methods are spliced to get the feature map with dimension of (1,1,2*C*). Because the channel number of the original input feature graph is *C*, we need to go through two convolution kernels with the size of 1×1 to reduce the dimension of the channel number to further extract the channel features. *R* represents the channel compression ratio, in this experiment, *R* = 16, the action process can be expressed as:

FC=Conv1×1(ReLU(BN(Conv1×1(concat[maxpool(X),avgpool(X)])))) (6)

where *F*_*C*_ is the channel attention output, *Conv*, ReLU, BN, maxpool and avgpool represents convolution operation, activation function, batch normalization, global maximum pooling and global average pooling.

#### Spatial attention module

**Fig 6:**
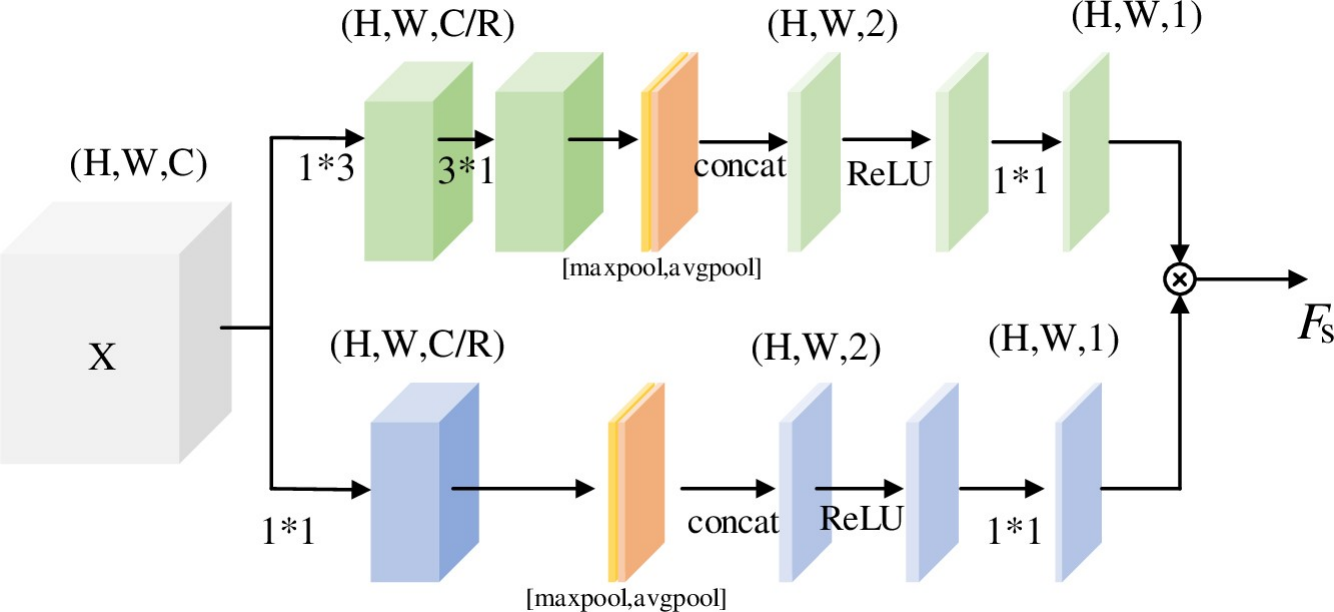
Spatial attention module.

Spatial attention focuses on the location information of the image, and removes the interference of background noise. For example, CBAM [23] adopts pooling channel compression in spatial branch. And Bottleneck Attention Module (BAM) [24] adopts serial convolution and hole convolution channel compression. In order to obtain more abundant spatial information, this paper uses different sizes of parallel convolution when compressing channels, as shown in Fig 6. Convolution kernels of 1×1 and 3×3 are used respectively to extract features to obtain rich feature information, in which convolution kernels of 3×3 are decomposed into 1×3 and 3×1. The maximum pooling and average pooling are used to aggregate the channel information on the two branches respectively. The channel number is compressed to 1 by convolution, and the information of the two branches is fused. The process of spatial attention module can be described as:

Fs=Conv1×1(ReLU(concat[maxpool(Conv3×1(Conv1×3(X)),avgpool(Conv3×1(Conv1×3(X)))]))×Conv1×1(ReLU(concat[maxpool(Conv1×1(X)),avgpool(Conv1×1(X))])) (7)

where *F*_*s*_ is the output of spatial attention module, *Conv*, ReLU, BN, maxpool and avgpool represents convolution operation, activation function, batch normalization, global maximum pooling and global average pooling.

## 4 Experiment

In this section, all kinds of the experimental settings will be introduced and the classification results of all related methods will be analyzed.

### 4.1 Datasets and training settings

We conducted experiments on three challenging fine-grained image classification datasets, namely CUB-200-2011 [12], Stanford dogs [13] and Stanford cars [14]. Table 2 summarizes the detailed statistics of datasets.

**Table 2:** Details of the three datasets.

Datasets	#Category	#Total	#Train	#Test
CUB-200-2011 [12]	200	11788	5994	5794
Stanford dogs [13]	120	20580	12000	8580
Stanford cars [14]	196	16185	8144	8041

The hardware configuration of the experiments: Intel Xeon e5-2683 V3 CPU, 32g running memory, single NVIDIA GTX 1080ti graphics card, 11g video memory. In win10 system, Pytorch framework is used as the experimental platform.

The input image is resized to 448 × 448. Each dataset is trained with 80 epochs and tested at the end of each epoch. The batch size is 8, the learning rate is 0.001, the momentum is 0.9, and the weight attenuation is set to 0.00001. SGD is used to train the loss function of the model. The pretrained parameters on ImageNet are used as the initial weight.

Classification accuracy is used to measure the performance of networks. It is expressed by:

$$A_{accuracy}=\frac{I_{a}}{I}$$ (8)

Where: *A*_*accuracy*_ represents the classification accuracy, *I*_*a*_ and *I* represent the number of correct classification and the total number of test images.

### 4.2 Experimental analysis

In order to verify that the proposed method can effectively improve the classification accuracy of fine-grained images, we conducted ablation studies and comparative analysis.

#### 4.2.1 Ablation studies

In order to explore the influence of different scale in multi-attention module, and try to find the best convolution combination, we use convolution combinations of different sizes to construct spatial attention module, including 1 × 1 and 3 × 3, 3 × 3 and 5 × 5, 5 × 5 and 7 × 7. The constructed attention module is tested on CUB-200-2011 data set, and the results are shown in Table 3.

**Table 3:** Top1 accuracy (%) of convolution combinations with different sizes on CUB-200-2011.

Convolution combination	Backbone	Top1(%)
1×1 and 3×3	ResNet50	**84.91**
3×3 and 5×5	ResNet50	84.68
5×5 and 7×7	ResNet50	84.35

From the Table 3, the convolution group with 1 × 1 and 3 × 3 can get the highest classification accuracy. We use the combination in the final multi-attention module.

To explore the effect of multi-attention module and pyramid convolution, WS-DAN is used as a benchmark. We conducted the following experiments on CUB-200-2011: 1) WS-DAN network with resnet50 as the backbone; 2) add the multi-attention module at the last layer of backbone; 3) add pyramid convolution to the backbone; 4) fuse the multi-attention with pyramid convolution. Table 4 shows the results of networks in different configurations. Compared with the baseline, for WS-DAN, using multi-attention module has 0.59% improvement. Using pyramid convolution can be improved by 1.31%. Fused module provides 1.60% improvement. In addition, using the channel group, our model has less computation cost than WS-DAN, which saves 0.08*10^7^ parameters and 0.27*10^9^ calculated numbers.

**Table 4:** Experimental results of ablation. Top1 accuracy (%) on CUB-200-2011.

Model	Backbone	Top1(%)	param	FLOPs
WS-DAN (baseline)	ResNet50	84.32	3.67×10^7^	4.13×10^9^
WS-DAN (att)	ResNet50+attention	84.91	3.79×10^7^	4.15×10^9^
WS-DAN(Py)	ResNet50+Pyconv	85.63	3.48×10^7^	3.85×10^9^
Ours (att + Py)	ResNet50+ attention +Pyconv	**85.92**	3.59×10^7^	3.86×10^9^

#### 4.2.2 Comparative analysis

**Fig 7:**
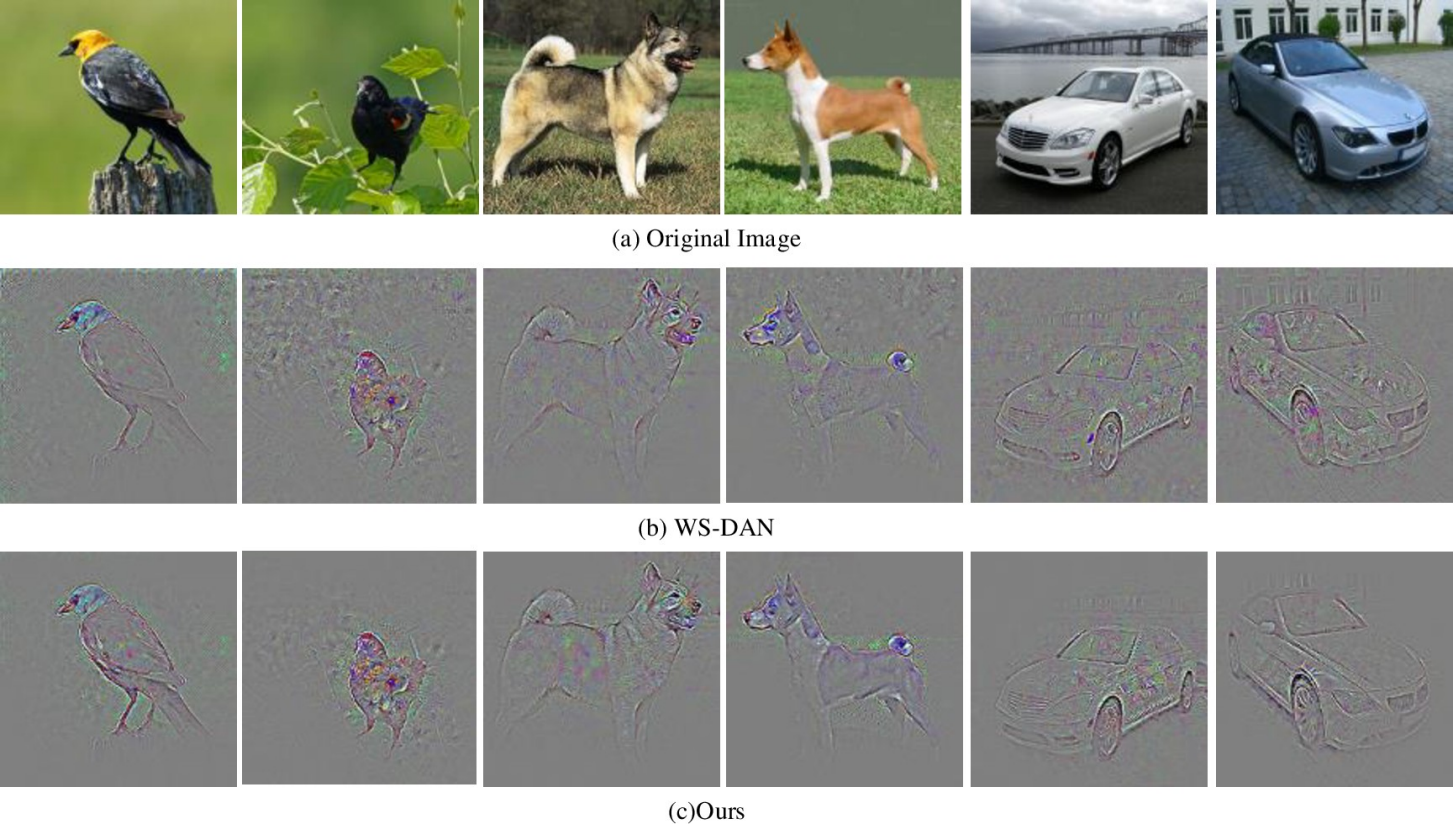
Back propagation saliency map.

**Fig 8:**
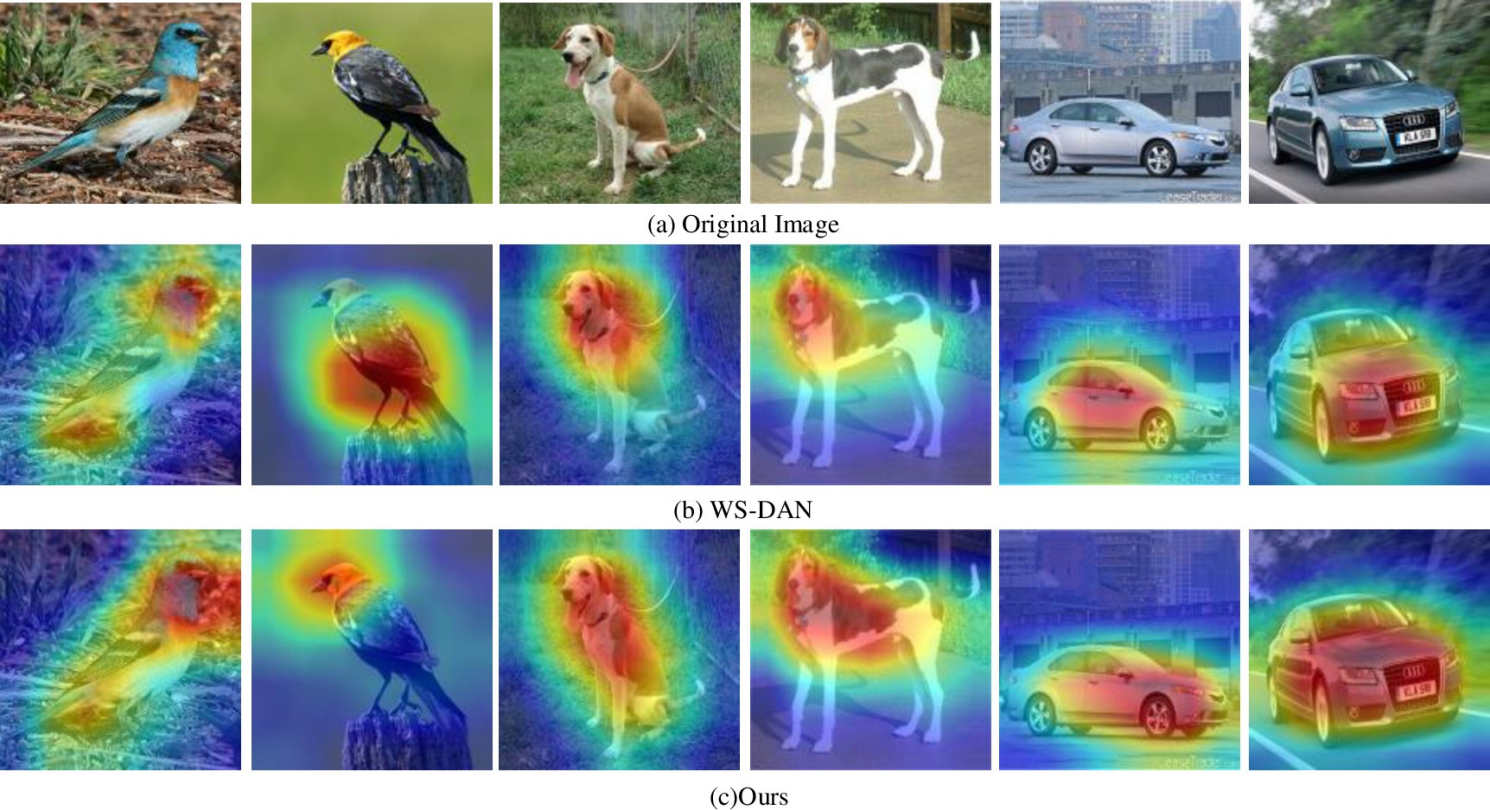
Visualization of attention.

*1) Comparison with WS-DAN model*. We improve the WS-DAN model based on the weakly supervised fine-grained algorithm, replacing the ordinary convolution kernel in resnet50 network with pyramid convolution kernel, and introducing the attention mechanism into the last layer of the network. The last layer of the original model and the proposed model is visualized respectively, in which Fig 7 is the back propagation saliency map, and Fig 8 is the visualization of attention.

Fig 7(A) is the original image. Fig 7(B) and 7(C) are the backpropagation saliency map of WS-DAN and the proposed method respectively. Compared Fig 7(B) and 7(C), WS-DAN has more background noise in feature extraction. The proposed method uses pyramid convolution kernel and multi-attention module to extract features and suppress the background noise effectively.

The visualization of attention is shown in Fig 8. Fig 8(A) is the original image, and Fig 8(B) and 8(C) are the visualization of the attention of WS-DAN and the proposed method. Different colors represent the different attention. And the deeper the red is, the higher the attention is. Comparing Fig 8(B) and 8(C), our method can locate the regions with distinguishing features more accurately, such as higher attention to the head of birds in the second column of Fig 8, which makes more computing resources incline to these key areas in the training and testing.

**Fig 9:**
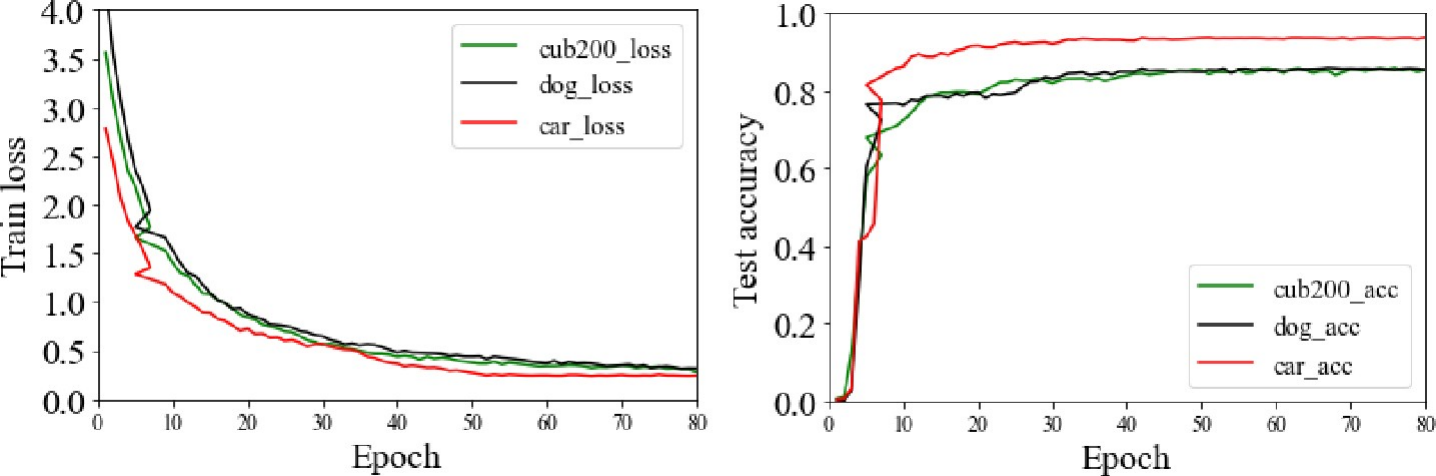
The test accuracy curve and train loss curve on CUB-200-2011, Stanford Cars and Stanford Dogs datasets.

Fig 9 is the curve of accuracy and loss. From Fig 9, we can see that the train loss of the proposed method on three datasets can be reduced steadily, which proves the rationality and universality of the proposed method. Because of the pretrained model, the test accuracy curve is rapidly improved in the first 10 epochs. Meanwhile, the test accuracy curve does not decline, which proves that there is no fitting phenomenon in this process.

*2) Comparison of different fine-grained algorithms*. We select resnet50 [25], BCNN [4], RA-CNN [5], MA-CNN [26], WS-DAN [8], and PMG [27] to compare with the proposed method, and test them on three public fine-grained data sets. The experimental results are shown in Tables 5 and 6. “1-Stage” indicates whether the training can be done in one stage. For one stage, the whole process of training and prediction is completed in the model, which does not need to be divided into multiple stages.

**Table 5:** Comparison results on CUB-200-2011.

Method	Backbone	1-Stage	Top1(%)
ResNet50 [25]	resnet50	√	73.01
BCNN [4]	vgg16	×	80.31
RA-CNN [5]	vgg19	×	83.27
MA-CNN [26]	vgg19	√	82.54
WS-DAN [8]	resnet50	√	84.32
PMG [27]	resnet50	√	85.31
Ours	pyconvresnet50	√	**85.92**

**Table 6:** Comparison results on Stanford Dogs and Stanford Cars.

Method	Top1(%)	
	Stanford Dogs	Stanford Cars
ResNet50	63.27	84.62
BCNN	79.36	88.29
RA-CNN	82.52	91.33
MA-CNN	81.73	90.57
WS-DAN	83.84	92.51
PMG	**-**	93.48
Ours	**85.82**	**93.64**

It can be seen from Tables 5 and 6 that the accuracy of the proposed method on CUB-200-2011, Stanford Dogs and Stanford Cars data sets reaches 85.92%, 85.82% and 93.64%. Compared with WS-DAN, the accuracy of these three datasets is improved by 1.60%, 1.98% and 1.13%. Compared with other methods, the proposed method also achieves the highest classification accuracy which further proves the effectiveness and universality of the proposed method.

## 5 Conclusion

In this paper, we design a new fine-grained classification network based on multi-scale pyramid convolution kernel. It adds pyramid convolution kernel into the main network. Through experiments, we find that pyramid convolution kernel has a better performance in extracting subtle features. In addition, a multi-attention module is designed, which includes spatial and channel attention. It has the advantages of lightweight and can be seamlessly connected in any CNN architecture. The experimental results show that the proposed method is better than other existing methods. However, the proposed method has still a large number of parameters. In the future, we will combine knowledge distillation to study the lightweight of fine-grained classification algorithm.

## Supporting information

S1 File (DOCX)
