# Supplementary material for: Fine-grained classification based on multi-scale pyramid convolution networks
Source: PLoS One. 2021 Jul 9;16(7):e0254054. doi: 10.1371/journal.pone.0254054 (PMC8270455; doi:10.1371/journal.pone.0254054)
Supplement: S1 File — (DOCX) [file pone.0254054.s001.docx]

**Supporting information**

We uploaded our minimal dataset to Figshare, which can be downloaded from the link: ( <https://figshare.com/s/3647e47f6b6ea4492fb6>)
